# Improved in vivo antitumor effect of a daunorubicin - GnRH-III bioconjugate modified by apoptosis inducing agent butyric acid on colorectal carcinoma bearing mice

**DOI:** 10.1007/s10637-016-0354-7

**Published:** 2016-05-05

**Authors:** Bence Kapuvári, Rózsa Hegedüs, Ákos Schulcz, Marilena Manea, József Tóvári, Alexandra Gacs, Borbála Vincze, Gábor Mező

**Affiliations:** National Institute of Oncology, Budapest, 1122 Hungary; MTA-ELTE, Research Group of Peptide Chemistry, Pázmány P. stny. 1/A, Budapest, 1117 Hungary; Department of Chemistry and Zukunftskolleg, University of Konstanz, 78457 Constance, Germany

**Keywords:** Colon cancer, Targeted cancer therapy, GnRH-III, Short chain fatty acids, Daunorubicin, In vivo tumor growth inhibition

## Abstract

Compared to classical chemotherapy, peptide-based drug targeting is a promising therapeutic approach for cancer, which can provide increased selectivity and decreased side effects to anticancer drugs. Among various homing devices, gonadotropin-releasing hormone-III (GnRH-III) peptide represents a suitable targeting moiety, in particular in the treatment of hormone independent tumors that highly express GnRH receptors (e.g. colon carcinoma). We have previously shown that GnRH-III[^4^Lys(Ac),^8^Lys(Dau = Aoa)] bioconjugate, in which daunorubicin was attached via oxime linkage to the ^8^Lys of a GnRH-III derivative, exerted significant in vivo antitumor effect on subcutaneously developed HT-29 colon tumor. In contrast, results of the study reported here indicated that this compound was not active on an orthotopically developed tumor. However, if Lys in position 4 was acylated with butyric acid instead of acetic acid, the resulting bioconjugate GnRH-III[^4^Lys(Bu),^8^Lys(Dau = Aoa)] had significant tumor growth inhibitory effect. Furthermore, it prevented tumor neovascularization, without detectable side effects. Nevertheless, the development of metastases could not be inhibited by the bioconjugate; therefore, its application in combination with a metastasis preventive agent might be necessary in order to achieve complete tumor remission. In spite of this result, the treatment with GnRH-III[^4^Lys(Bu),^8^Lys(Dau = Aoa)] bioconjugate proved to have significant benefits over the administration of free daunorubicin, which was used at the maximum tolerated dose.

## Introduction

Colorectal cancer is the third most common type of cancer worldwide, with nearly 1.4 million new cases diagnosed in 2012. It is predicted that the number of cases will rise to 2.4 million by 2035. About 54 % of colorectal cancer cases occur in more developed countries, with the highest incidence rate in Europe [1]. Thus, in addition to colon cancer prevention by changing nutritional habits [2], the development of efficient therapeutic strategies is of utmost importance. Peptide-based targeted tumor therapy, which has been investigated in the last decades, might be an effective targeted chemotherapeutic approach to cure colon cancer as well [3]. Nevertheless, only a few research reports have been published in this field yet [4].

The principle of targeted tumor therapy relies on the structural and/or functional differences between cancer cells and healthy ones [5]. One of the possible targeted chemotherapeutic approaches is based on the attachment of an anticancer drug to a targeting moiety, which recognizes tumor specific receptors or cell surface structures that are highly expressed on tumor cells [6]. The homing device conjugated to the chemotherapeutic agent enables the specific binding to the tumor cell surface, without affecting the healthy tissues. Such targeting moieties could be hormone peptides (e.g. gonadotropin releasing hormone (GnRH), somatostatin, bombesin), whose receptors are highly expressed on many types of tumors, while their presence on healthy tissues is limited [7].

**Fig. 1:**
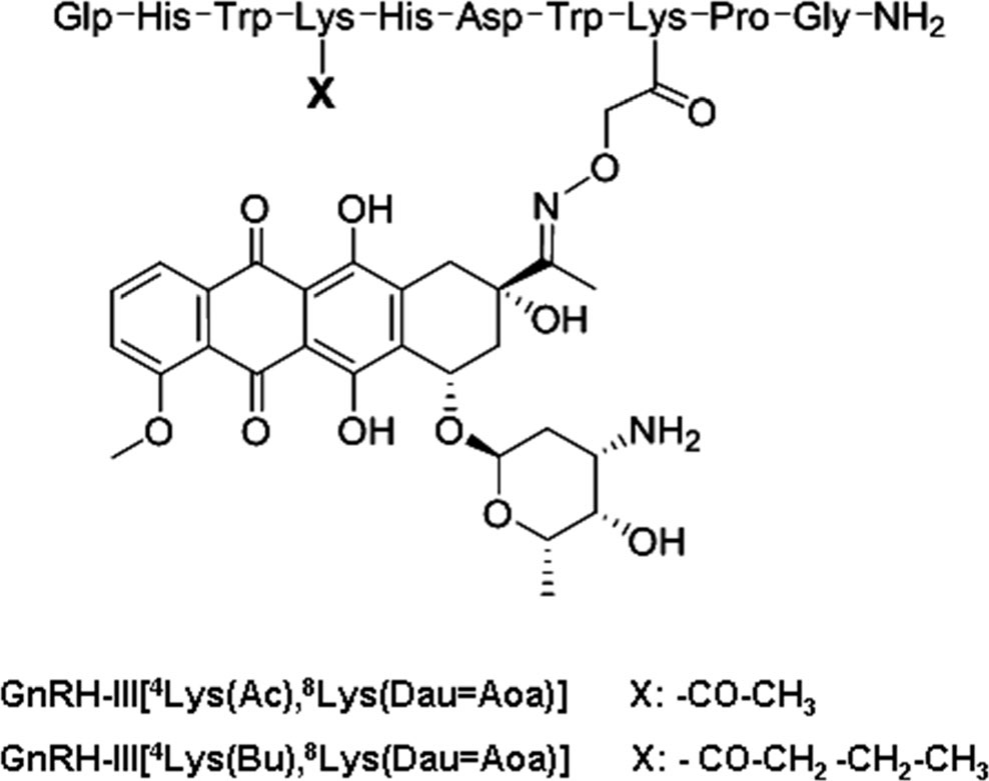
Structure representation of daunorubicin-GnRH-III derivative bioconjugates

In our research, lamprey GnRH-III (Glp-His-Trp-Ser-His-Asp-Trp-Lys-Pro-Gly-NH_2_, where Glp is pyroglutamic acid) has been employed as a key molecule for drug targeting [8]. It has been found that GnRH-III binds to tumoral GnRH receptors (GnRH-R), while its endocrine effect in mammals is marginal [9, 10]. Thus, hormonal side effects in case of targeted tumor therapy using GnRH-III as a homing device are expected to be insignificant, this feature being important in the treatment of hormone independent tumors like colorectal cancer. Considering that lysine in position 8 of GnRH-III can be used for drug conjugation, this amino acid has been used in our previous studies for the attachment of daunorubicin (Dau) via oxime linkage. For this purpose, the side chain of Lys was modified with an aminooxyacetyl (Aoa) group [11]. Aoa was coupled to the ε-amino group either directly or through a GFLG tetrapeptide spacer cleavable by Cathepsin B, enzyme known to be overexpressed in cancer cells. These bioconjugates exerted significant antitumor activity both in vitro and in vivo. Moreover, no signs of toxicity were detected on BDF-1 healthy female mice at a dose up to 30 mg Dau content/kg [12]. Murine C26 colon cancer bearing Balb/c mice (subcutaneously implanted tumors) were treated with the bioconjugates at a dose of 15 mg Dau content/kg. Compared to the untreated control group, longer survival and about 30–40 % tumor growth inhibition could be achieved. The in vivo antitumor effect of the bioconjugates was more pronounced on human HT-29 colon cancer bearing SCID mice (*s.c.* implanted tumor), the tumor growth inhibition being 40–50 % in this case [12]. However, if the C26 tumor was orthotopically developed in Balb/C mice, the effect of the bioconjugates on tumor growth was lower than 10 %. Therefore, more potent analogues were developed [13]. It has previously been reported that Ser in position 4 could be exchanged by Lys or Lys(Ac) without losing the antitumor activity of GnRH-III analogs [14]. The replacement of Ser by Lys(Ac) in the Dau containing bioconjugate led not only to increased in vitro and in vivo antitumor effect, but also to enhanced enzymatic stability and cellular uptake. The GnRH-III[^4^Lys(Ac),^8^Lys(Dau = Aoa)] bioconjugate exerted 50 % growth inhibition of orthotopically developed C26 tumor. This promising result prompted us to further develop short chain fatty acid containing derivatives [15]. The rational of this drug design strategy was that short-chain fatty acids (SCFAs), in particular butyrate, which is produced by anaerobe bacterial fermentation of dietary fiber within the large colon, are known for their potential to act as chemopreventive agents by slowing the cell growth and activating apoptosis in colon cancer cells [16]. From all prepared SCFA-modified bioconjugates, the most potent one on HT-29 human colon cancer cells was GnRH-III[^4^Lys(Bu),^8^Lys(Dau = Aoa)], in which Lys in position 4 was acylated with butyric acid (Fig. 1). Its IC_50_ value was 2.2 ± 0.6 μM, while this value was 7.4 ± 2.6 μM in the case of acetylated version. The reasons for the higher in vitro antitumor effect of the butyrated bioconjugate were extensively discussed in one of our previous reports [15]. In brief, it was found that both compounds, similarly to the other SCFA containing bioconjugates, were rather hydrophilic and no significant difference between their octanol-water partition coefficient and membrane permeability could be determined using lipid Langmuir monolayer. Therefore, secondary structure analysis by circular dichroism (CD) spectroscopy was further performed. However, the CD spectra of the bioconjugates did not show any difference. The binding of the bioconjugates to the GnRH receptor was also investigated and indicated higher affinity in the case of the butyrated bioconjugate. It has been reported that the N- and C-terminal domains of GnRH derivatives play an important role in the receptor binding. In contrast to the U-shape of GnRH-I, GnRH-III adopts an extended structure resulting in lower binding affinity to the type I GnRH receptor [17]. We assume that the replacement of Ser in position 4 by an acylated lysine provides a more suitable structure for receptor binding, which is stabilized more by a larger fatty acid chain, such as butyric acid. However, the fatty acid chain should not be too large, so that to negatively affect the binding of the N-terminus to the receptor. This is a possible explanation why the modification of ^4^Lys with butyric acid provides optimal features in the case of oxime bond-linked Dau-GnRH-III bioconjugates. Furthermore, we demonstrated by LC-MS that the apoptosis inducing agent butyric acid was released from the bioconjugate in the presence of lysosomal homogenate. It was also indicated that the oxime bond-linked daunorubicin-GnRH-III derivative bioconjugates are stable for at least 24 h in human serum [13, 18]. On the basis of these results, the in vivo tumor growth inhibitory effect of GnRH-III[^4^Lys(Bu),^8^Lys(Dau = Aoa)] has further been evaluated on colon carcinoma bearing mice, in comparison with that of GnRH-III[^4^Lys(Ac),^8^Lys(Dau = Aoa)] and the results are reported here.

## Materials and methods

### Daunorubicin-GnRH-III derivative bioconjugates

Daunorubicin-GnRH-III derivative bioconjugates were prepared by a combination of solid phase peptide synthesis and chemoselective ligation (e.g. oxime bond formation) as previously described [13, 15]. The bioconjugates were purified by RP-HPLC using a mixture of 0.1 % TFA/water and 0.1 % TFA/MeCN-water (80:20, *v*/v). The freeze-dried bioconjugates, without changing the tfa counter ions, were used in the in vivo studies, in order to evaluate their tumor growth inhibitory effect on colon carcinoma bearing mice.

### NSG mice

The immunodeficient NSG (**N**on Obese Diabetic **S**evere Combined ImmunoDeficient interleukine **G**amma receptor chain knock-out) (NOD.Cg-*Prkdc*^*scid*^*Il2rg*^*tm1Wjl*^/SzJ) mice were originated from Jackson Laboratories. The mice were held in filter-top boxes in the experimental barrier rooms and every box-opening was done under a Class 100 laminar-flow hood by an operator dressed in sterilized surgical attire. The animal housing density was in accordance with the international recommendations. The cage components, corn-cob bedding and food (VRF1 from Special Diet Services) were steam-sterilized in autoclave (121 °C, 20 min). The distilled water was acidified to pH 3 with hydrochloric acid.

The animals used in these studies were cared for according to the “Guiding Principles for the Care and Use of Animals” based upon the Helsinki declaration, and they were approved by the local ethical committee. Our permission for breeding and performing experiments with laboratory animals is valid until 2020/10/12 (registration numbers: 22.1/772/3/2010 and PEI/001/2574–6/2015).

### Study of the chronic toxicity

NSG adult male mice, weighing 26–34 g, were used. Daunorubicin was dissolved in distilled water at a concentration that allowed the dose to be given in a volume of 0.1 mL/10 g body weight. In this experiment, each group consisted of 3 mice, which were treated by intraperitoneal (*i.p*.) administration on days 1, 4, 8, 12 and 16. The toxicity was assessed on the basis of lifespan and body weight and the survival time was followed for 20 days.

### Development of the primary tumor

Female NSG mice, weighing 24–28 g, were used in this study. The xenografts were established by subcutaneous injection of ca. 10^6^ HT-29 (GnRH receptor positive) human colon carcinoma cells, which were from ATCC and cultured according to their standards. The mice with palpable tumors were killed by cervical-dislocation, disinfected with iodine, and the subcutaneous tumor was dissected out aseptically. Tumor pieces of 2–3 mm^3^ were transplanted under aseptic conditions, orthotopically, into narcotized NSG mice.

### Tumor transplantation

For direct implantation, mice were anesthetized (narcotic mixture: tiletamine, zolazepam, xylazine, butorphanol) and the abdomen was sterilized with iodine and alcohol swabs. A small midline incision (0.5 cm) was made and the colorectal part of the intestine was exteriorized. Serosa of the site where the tumor pieces were to be implanted was removed. A piece of 2 mm^3^ HT-29 human colon tumor was implanted on the top of the animal intestine; an 8/0 surgical (polypropylene) suture was used to suture it on the wall of the intestine. The intestine was returned to the abdominal cavity and the abdominal wall was closed with 4/0 surgical (polyglycolic acid) sutures. The wound was sterilized with iodine and alcohol swab again and the animals were kept in a sterile environment. For pain release and rehydration, mice were treated *s.c.* with 100 mg/kg algopyrine in Ringer solution containing 5 % glucose. On the next day, no signs of pain and stress of mice were observed.

### Doses and treatments

Based on the results of the chronic toxicity study, we used only 1 mg (1.773 μmol)/kg free Dau, weekly, in the Dau treated group. Thirteen treatments were applied from the day 5 after tumor transplantation until the day 50 in the groups treated with the bioconjugates (every Monday and Thursday). The treatments were performed by *i.v.* (intravenous) or *i.p.* administration; the compounds were dissolved in distilled water (0.1 mL/10 g body weight).

In this experiment, 7 mice/group were used for the treatment with the bioconjugates (GnRH-III[^4^Lys(Ac),^8^Lys(Dau = Aoa)] and GnRH-III[^4^Lys(Bu),^8^Lys(Dau = Aoa)]) as well as in the control group.

The bioconjugates were administered at different doses during the treatment period. In the first five treatments, they were *i.v.* administered, at a dose of 28.435 μmol/kg body weight (15 mg Dau content in the bioconjugate/kg). From the 6th to the 13th treatment, they were *i.p.* administered at a dose of 14.217 μmol/kg body weight (7.5 mg Dau content in the bioconjugate/kg). The first five doses represented the initial round of treatments, which was followed by the maintenance therapy. In the maintenance treatments, 5 % glucose solution instead of distilled water was used, in order to minimize the harmful effects of tfa salts that were present in the lyophilized bioconjugates.

The mice from all groups were sacrificed by deadly overslept cocktail (0.3 mL/mouse concentrated nembutal (pentobarbitone) solution), on day 50 after tumor transplantation.

After that, the tumors were removed and the tumor weight was determined in all cases.

### Statistical analysis

The statistical analyses were performed by Medcalc® version 12.1.3.0. (Broekstraat 52, B-9030 Mariakerke, Belgium) using the non-parametric Mann-Whitney (independent samples) test. The experimental data were filtered by Gaussian statistics g. *P*-values lower than 0.05 were considered statistically significant.

### Routine histological examination

The removed and fixed tissue samples were dehydrated in a graded series of ethanol, infiltrated with xylene and embedded into paraffin. Histological sections were stained with Harris hematoxylin and eosin (10:1, *v*/v, 1 % solutions) in acidified 70 % ethanol and mounted. The histological samples were examined under a light microscope (Olympus CH30, Olympus Optical, Japan).

### Determination of the proliferative index and vascularization in orthotopic HT-29 tumor tissues

Before ending the experiment (30 min prior to sacrificing the mice), the tumor bearing animals were injected *i.p.* with 5-bromo-2 V-deoxyuridine (BrdU, 200 mg/kg; Sigma-Aldrich Kft, Budapest, Hungary). After 6 h, 5–7 μm thick tissue sections were excised from the newly frozen tumors and used for the detection of BrdU positive cells using an anti-BrdU monoclonal antibody (Becton Dickinson Hungary Kft., Budapest, Hungary), according to the manufacturer’s protocol. Positive cells were visualized with TRITC-conjugated anti-mouse IgG (1:100, Sigma). Endothelial cells were distinguished from tumor cells by treating the samples with rat anti-mouse CD31 antibody and labelled with biotinylated anti-rat IgG and streptavidin-FITC (Vector Laboratories, Burlingame, CA). The nuclei were stained with the Hoechst 33,342 dye (Molecular Probes, Eugene, OR). The number of labelled HT-29 tumor cells and the quantity of CD31-positive samples were determined in two different tumors by microscopic analysis of 6 independent regions. The vascularization extent of primary tumors was determined by microscopic analysis of two different living tumors, where four independent areas of living cells were counted and the percentage of separated endothelial cells covering a 10× magnification field of vision was calculated [12].

## Results

### Chronic toxicity of daunorubicin on NSG healthy mice

**Fig. 2:**
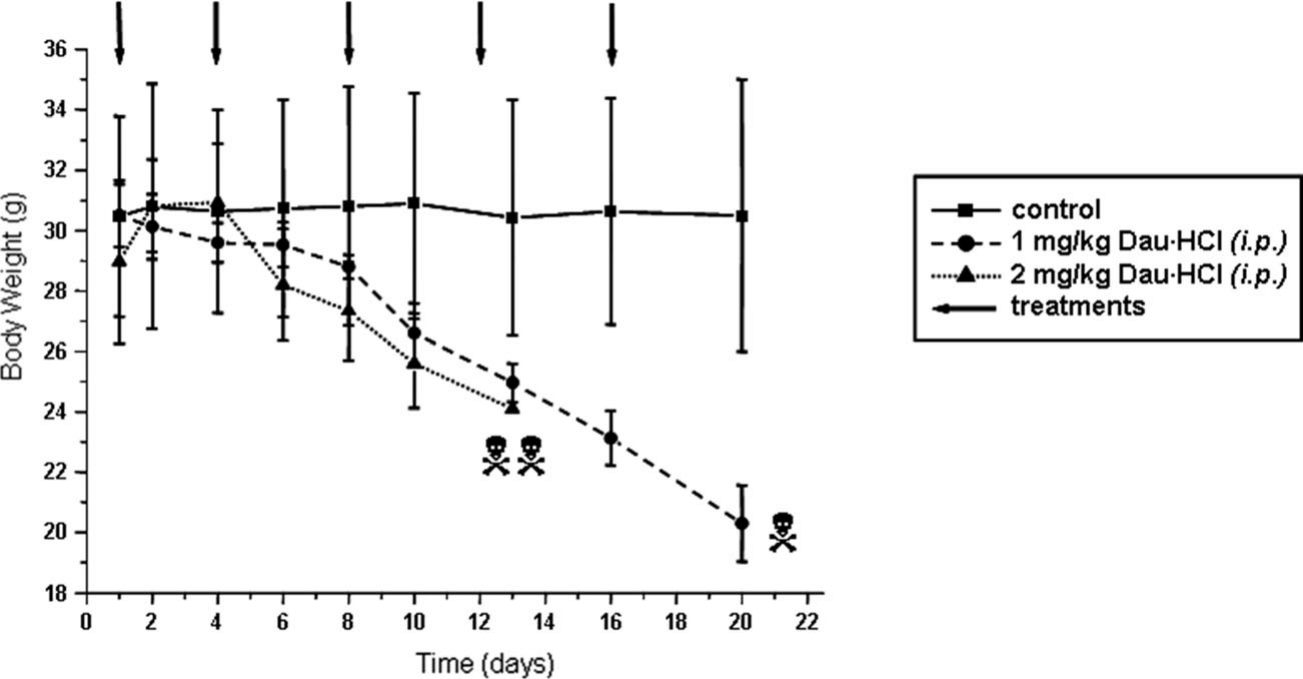
Chronic in vivo toxicity of daunorubicin on NGS mice

Before starting the treatment of tumor bearing mice, the tolerable dose of daunorubicin was tested on NSG healthy mice. Daunorubicin hydrochloride was *i.p.* administered at a dose of 1 or 2 mg/kg body weight, on days 1, 4, 8, 12 and 16. In both cases, a significant loss of weight of the animals was observed. Furthermore, 2 of 3 mice died on day 13 after the fourth treatment in the group treated with 2 mg/kg dose. The first animal died in the group treated with 1 mg/kg dose on day 20, when the experiment was stopped. The body weight of the animals decreased from ~30 g to ~20 g during this time (Fig. 2). Therefore, we decided to administer daunorubicin once a week at a dose of 1 mg/kg.

### Tumor growth inhibition

HT-29 human colon tumor (2 mm^3^) was orthotopically implanted on the intestine of mice. The beginning of tumor growth was controlled on two sacrificed animals and the treatments with the free drug and bioconjugates started on day 5 after tumor implantation.

**Fig. 3:**
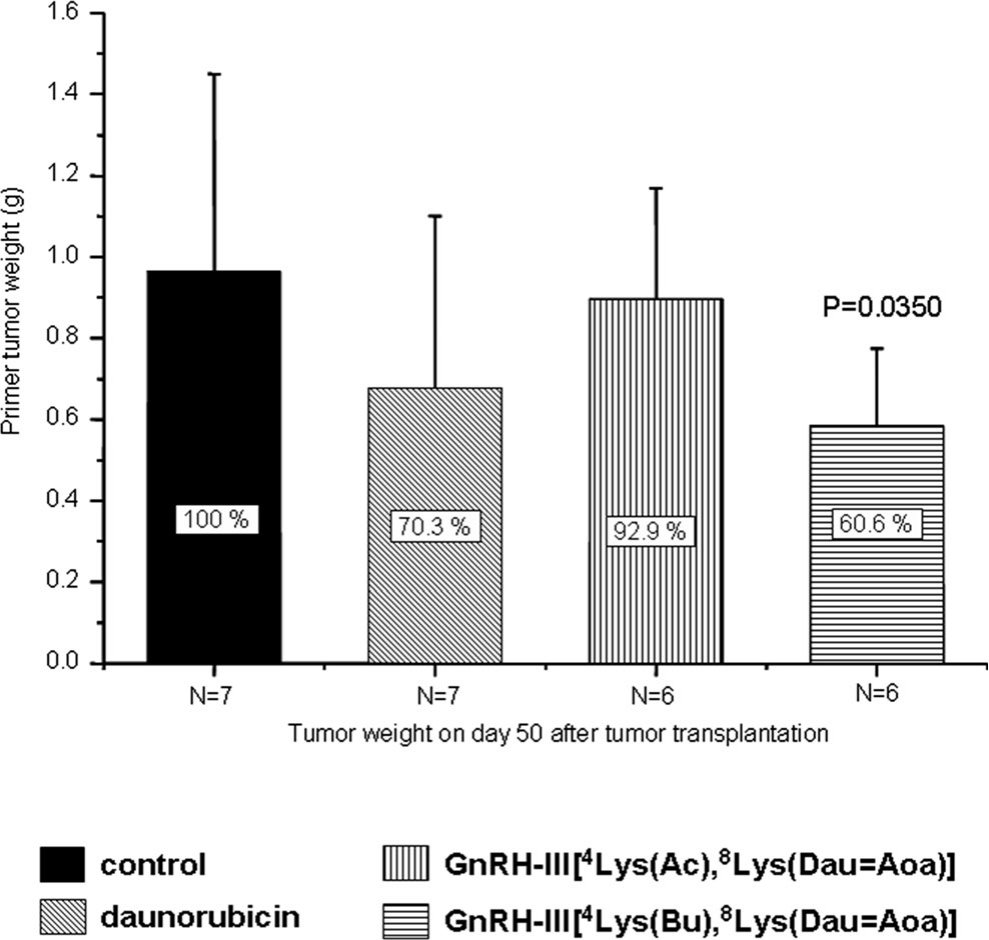
In vivo antitumor effect of Dau-GnRH-III derivative bioconjugates and free daunorubicin on HT-29 orthotopic primary human colon carcinoma (Mean ± SD, Mann-Whitney test)

Mice were treated *i.v.* with daunorubicin once a week at a dose of 1 mg (1.773 μmol)/kg. Seven injections were carried out until the day 50, when the study was finished. The bioconjugates were administered twice a week. The first five injections represented the initial round of treatments using 28.435 μmol/kg body weight (15 mg Dau content in the bioconjugate/kg), while half of this amount (14.217 μmol/kg body weight; 7.5 mg Dau content) was applied in further eight administrations as maintenance therapy. After the fifth *i.v*. injection with the bioconjugates, the tail inflammation was observed; therefore, the treatments continued by *i.p.* administration. The mice were sacrificed on the day 50 after tumor implantation and the tumors were removed, followed by measuring their weight (Fig. 3). In comparison with the control group (7 animals), about 30 % tumor growth inhibition was observed in the group (7 animals) treated with free daunorubicin. In contrast to our previous study in which *s.c.* implanted C26 tumor was used, the GnRH-III[^4^Lys(Ac),^8^Lys(Dau = Aoa)] bioconjugate exhibited very moderate, not significant tumor growth inhibition (only ~7 % to the control group). In contrast, the GnRH-III[^4^Lys(Bu),^8^Lys(Dau = Aoa)] bioconjugate exerted a significant inhibitory effect on tumor growth, leading to ~40 % inhibition in average compared to the control (Fig. 3).

### Liver- and cardiotoxicity of free daunorubicin and its GnRH-III derivative bioconjugates

**Fig. 4:**
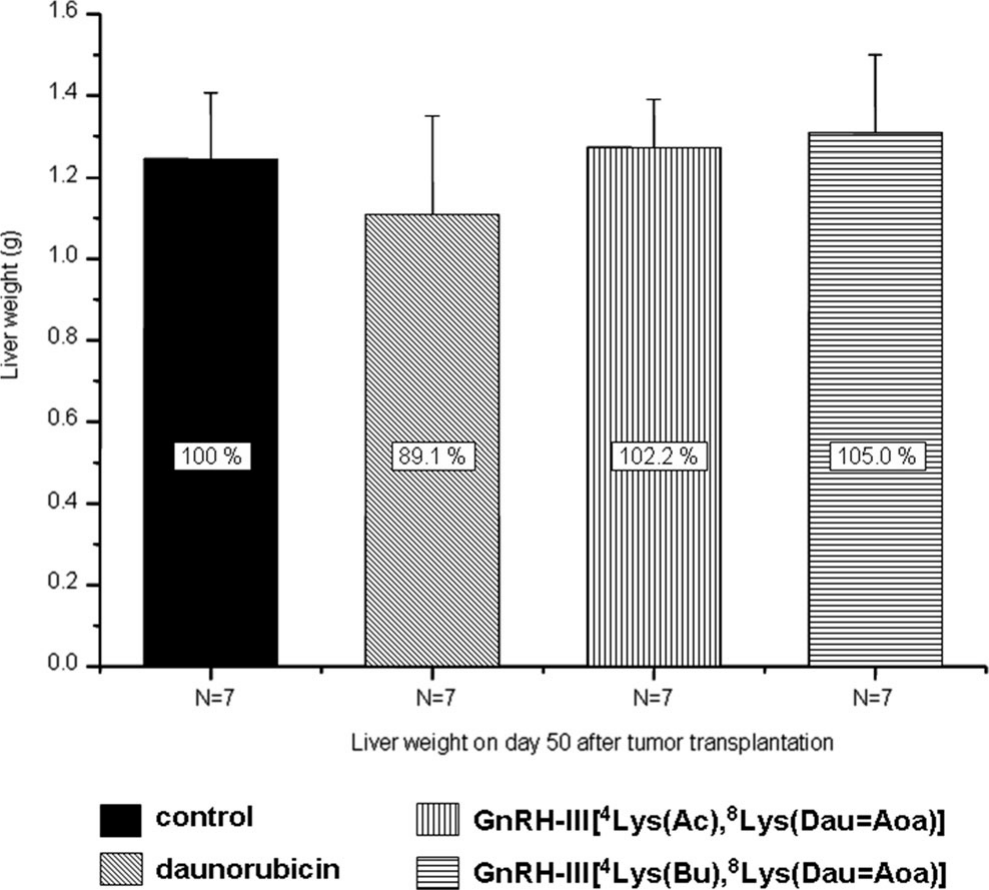
Macroscopic liver toxicity of Dau-GnRH-III derivative bioconjugates and free daunorubicin (Mean ± SD)

Macroscopic toxic side effects of daunorubicin and its GnRH-III derivative bioconjugates were assessed after completing the treatments of mice. The liver toxicity was evaluated by determining the weight of the liver. The average liver weight of mice treated with the free drug decreased with about 11 %, difference which was not significant to the control group. Slight liver weight enhancement (2 % and 5 % in case of the acetylated and butyrated bioconjugates, respectively) was observed after the treatments with the bioconjugates (Fig. 4). The cardiotoxicity was assessed on the basis of histological examination using hematoxylin and eosin stain of tissue slides [19]. Considering that no changes indicating heart failure were observed by light microscopy, in comparison with the control group, further studies of other biomarkers for heart diseases were not investigated. Similar to the liver, in any of the treated animals there was no macroscopically observed tissue damage of heart that would indicate cardiotoxicity. Therefore, we could conclude that neither the bioconjugates nor the free daunorubicin showed any liver- or cardiotoxicity at the applied doses.

### Macroscopic metastases in treated and untreated mice

The tumor metastases on different organs (liver, lung, spleen, lymph node, pancreas, diaphragm and kidney) were also investigated in the present study. The results summarized in Table 1 indicated that neither the free drug nor the bioconjugates had significant influence on the global metastasis. However, the tumor was less spread out on several organs in the case of treated animals compared to the untreated ones. Liver metastasis was observed in each case. The lung was also invaded by tumors almost in all tested animals. The observed metastases on spleen were not significant in any group. Significant improvement in preventing metastasis was detected on pancreas and lymph nodes in the treated groups compared to the control. The improvement on the latter one was more pronounced when the animals were treated with the bioconjugates. However, the diaphragm was more affected by tumors, particularly in the case of GnRH-III[^4^Lys(Ac),^8^Lys(Dau = Aoa)] treated mice.

**Table 1:** Metastasis on different organs

Groups (7 animals in each group)	Liver	Lung	Spleen	Pancreas	Lymph node	Diaphragm
Control	7/7 100 %	6/7 85.7 %	1/7 14.3 %	2/7 28.6 %	5/7 71.4 %	2/7 28.6 %
Daunorubicin	7/7 100 %	7/7 100 %	0/7 0 %	0/7 0 %	4/7 57.2 %	3/7 42.9 %
GnRH-III[^4^Lys(Ac),^8^Lys(Dau = Aoa)]	7/7 100 %	6/7 85.7 %	1/7 14.3 %	0/7 0 %	2/7 28.6 %	5/7 71.4 %
GnRH-III[^4^Lys(Bu),^8^Lys(Dau = Aoa)]	7/7 100 %	7/7 100 %	1/7 14.3 %	0/7 0 %	3/7 42.9 %	3/7 42.9 %

### Tumor proliferation and vascularization

**Fig. 5:**
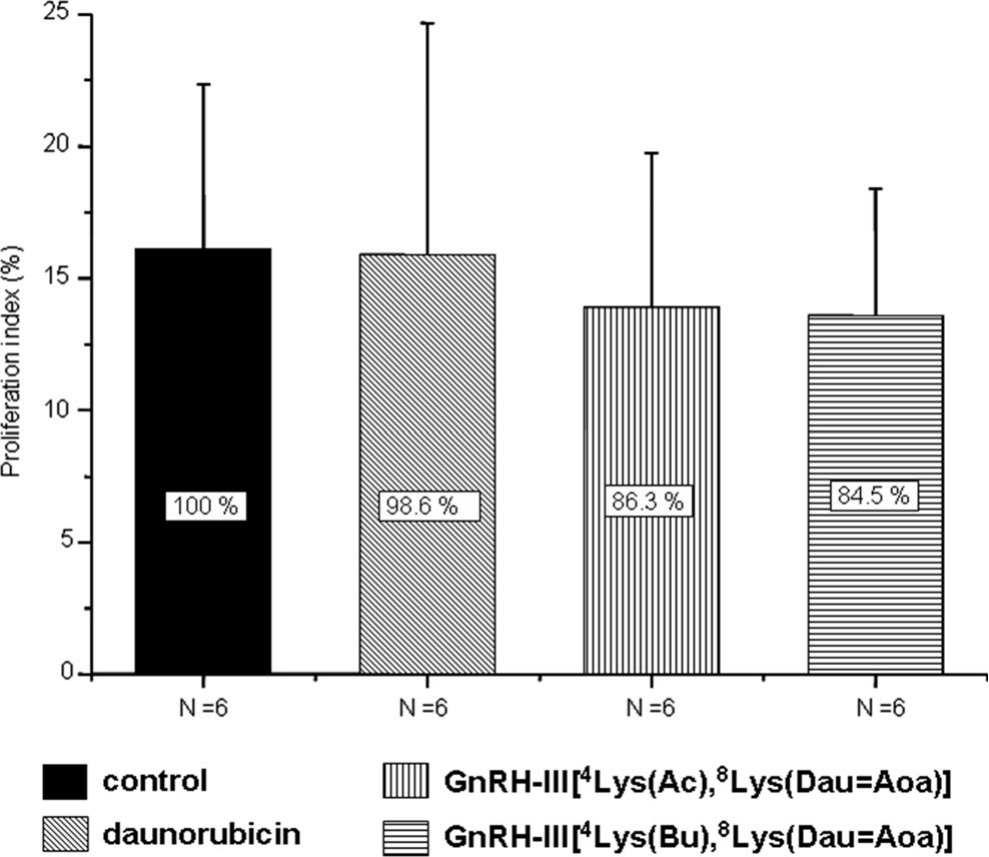
Proliferation index of HT-29 orthotopic primary human colon carcinoma treated with Dau-GnRH-III derivative bioconjugates or daunorubicin (Mean ± SD)

Compared to the control animals, the proliferation rate of orthotopically developed HT-29 tumors did not significantly decrease in the groups treated with the bioconjugates (~15 %) (Fig. 5). Nevertheless, this effect was not observed at all on Dau treated animals.

**Fig. 6:**
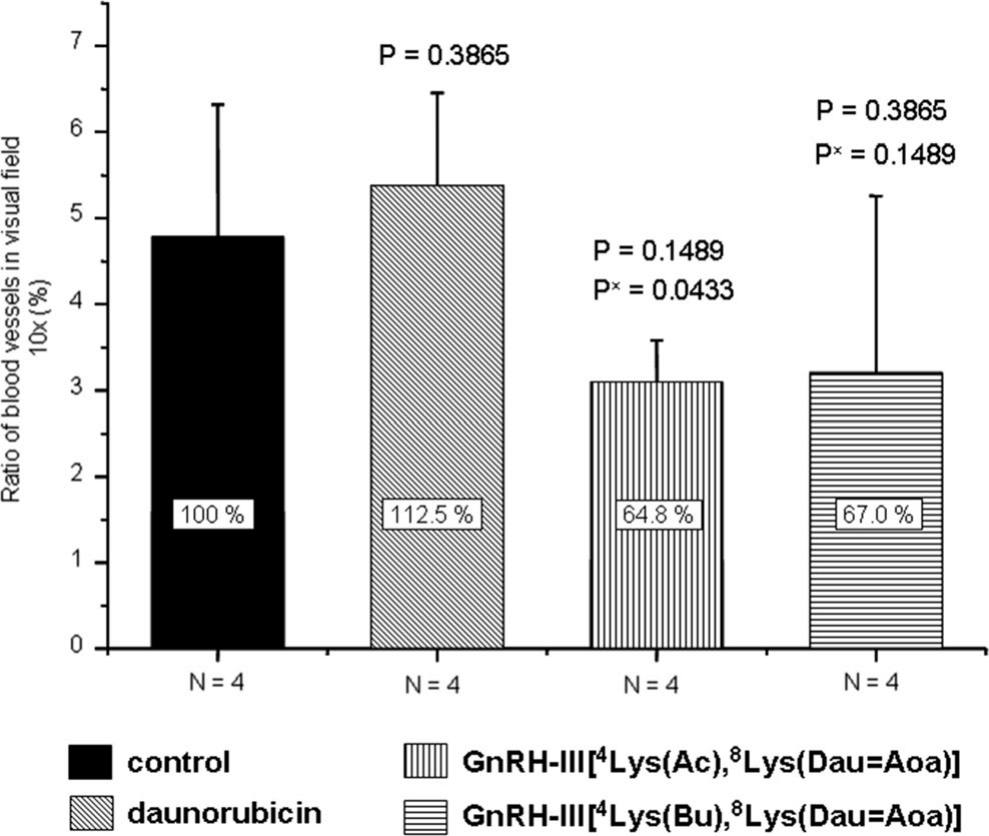
Vascularization of HT-29 orthotopic primary human colon carcinoma treated with Dau-GnRH-III derivative bioconjugates or daunorubicin (Mean ± SD, Whitney test) (P^x^ = free Dau treated group vs. bioconjugate treated group)

In contrast to the proliferation index, the tumor vascularization (vessel density) in mice treated with the bioconjugates was considerable lower (ca. 35 %) compared to the controls (Fig. 6). Furthermore, the treatment with the free drug increased the number of newly formed blood vessels.

## Discussion

Colon cancer is one of the most widespread types of cancer in developed countries; therefore, development of efficient treatment procedures is necessary. Targeted tumor therapy is a promising approach to treat different types of cancer without significant side effects. Among homing devices that might be used for drug targeting, hormone peptides like GnRH or somatostatin are highly promising. These peptides have own antiproliferative effect, which could enhance the activity of the targeted drug molecules. At present, some of these bioconjugates are under clinical investigations [20].

In our research, GnRH-III peptide was mainly employed as a targeting moiety, due to its low endocrine effect in mammals and significant antiproliferative effect on different types of cancer cells. Our previous results showed that bioconjugates in which daunorubicin was attached to the side chain of Lys in position 8 via oxime linkage exerted significant antitumor effect both in vitro and in vivo*.* However, the efficacy of the bioconjugates was highly dependent on the tumor type and whether this was subcutaneously or orthotopically developed in mice. Orthotopically developed tumors represent the native conditions much better than the subcutaneously implanted ones. In this case, also the tumor metastases and the vascularization of primary tumors could occur easier than those of isolated *s.c.* implanted tumors. Therefore, in the present study, HT-29 human colon tumor was implanted on the intestine of immunodeficient NSG mice. Two Dau-GnRH-III derivative bioconjugates that showed high antitumor activity in vitro were selected for the treatment. These compounds contained a GnRH-III derivative in which Ser in position 4 was modified by Lys acylated with acetic or butyric acid (Fig. 1). These bioconjugates were not toxic on mice up to 30 mg daunorubicin content/kg body weight. However, the maximum tolerated dose of free daunorubicin was 1–2 mg/kg/week in immunodeficient tumor bearing mice. According to the in vivo chronic toxicity study, we used only 1 mg/kg free Dau, weekly. In contrast to this, the bioconjugates were administered two times per week, at a dose of 15 mg Dau content in bioconjugate/kg, five times as initial treatment and then 7.5 mg for the maintenance treatment. The results of this study indicated that the acetylated version of Dau-GnRH-III bioconjugate had no effect on this type of tumor (Fig. 3). Interestingly, it has previously shown significant antitumor effect (50 % tumor growth inhibition) on an orthotopically developed C26 mouse colon tumor [14]. A possible explanation of this result might be the difference in the doubling time of cancer cells. The C26 tumor is an aggressive and fast proliferating tumor type, while HT-29 grows much slower. In contrast to this, the butyric acid modified bioconjugate exerted higher tumor growth inhibition than the free daunorubicin (40 % and 30 % to the control, respectively). The increased tumor growth inhibition of GnRH-III[^4^Lys(Bu),^8^Lys(Dau = Aoa)] compared to GnRH-III[^4^Lys(Ac),^8^Lys(Dau = Aoa)] might partially be explained by the higher receptor binding affinity and cellular uptake of the butyrated version, as determined in previous in vitro studies [15].

The toxic side effects of daunorubicin and bioconjugates on different organs were also evaluated. Particular interest was in determining the liver and heart toxicity, because of the well known cardiotoxity of anthracyclines. Except for a moderate (11 %) liver shrinkage in the case of daunorubicin treatment (Fig. 4), neither the bioconjugates nor the free daunorubicin showed any toxicity at the applied doses.

The number of dividing cells in tumor tissues as well as the tissue vascularization are important factors of tumor growth. Therefore, the proliferative index was calculated and the newly formed blood vessels in the tumor were counted. In the case of mice treated with the bioconjugates, the proliferative index was lower than that of control animals and mice treated with the free drug. However, the decrease was not significant. Interestingly, the number of blood vessels was considerably lower in the case of mice treated with the bioconjugates (64–67 % to the control), while a slightly increased number was observed in the case of daunorubicin treated animals (112 %). This might explain the higher tumor growth inhibition induced by the bioconjugate and could be an important aspect in its beneficial effect over the application of the free drug.

In conclusion, we clearly showed that GnRH-III[^4^Lys(Bu),^8^Lys(Dau = Aoa)] bioconjugate, in which daunorubicin was attached via a stable oxime linkage to ^8^Lys and Ser in position 4 of the homing peptide was replaced by Lys(Bu), provided benefits over the free drug in the treatment of colon carcinoma. This bioconjugate exerted significantly higher tumor growth inhibitory effect on orthotopically developed human HT-29 colon cancer bearing mice than the free Dau at the maximum tolerated dose. Furthermore, the bioconjugate slightly decreased the tumor proliferation index and reduced the vascularization of tumor tissues at a high level. The bioconjugate did not show toxic side effects; however, similarly to the free drug it was not able to prevent metastases on different organs.

Taken together these results, we can conclude that GnRH-III[^4^Lys(Bu),^8^Lys(Dau = Aoa)] bioconjugate is a promising candidate for targeted chemotherapy of colon cancer. However, its combination with a metastasis preventing agent might be necessary and should be investigated in future studies.
